# Preparation of Human Milk Fat Substitutes: A Review

**DOI:** 10.3390/life12020187

**Published:** 2022-01-27

**Authors:** Xuan Jiang, Xiaoqiang Zou, Zhonghao Chao, Xiuli Xu

**Affiliations:** 1 Provincial Key Laboratory of Cereal Resource Transformation and Utilization, Henan University of Technology, Zhengzhou 450001, China; jiangxuan@stu.jiangnan.edu.cn; 2 National Engineering Research Center for Functional Food, Collaborative Innovation Center of Food Safety and Quality Control in Jiangsu Province, National Engineering Laboratory for Cereal Fermentation Technology, State Key Laboratory of Food Science and Technology, School of Food Science and Technology, Jiangnan University, 1800 Lihu Road, Wuxi 214122, China; 6200113152@stu.jiangnan.edu.cn (Z.C.); 6200112092@stu.jiangnan.edu.cn (X.X.)

**Keywords:** human milk, human milk fat substitutes, structured lipids, infant formula

## Abstract

Human milk is generally regarded as the best choice for infant feeding. Human milk fat (HMF) is one of the most complex natural lipids, with a unique fatty acid composition and distribution and complex lipid composition. Lipid intake in infants not only affects their energy intake but also affects their metabolic mode and overall development. Infant formula is the best substitute for human milk when breastfeeding is not possible. As the main energy source in infant formula, human milk fat substitutes (HMFSs) should have a composition similar to that of HMF in order to meet the nutritional needs of infant growth and development. At present, HMFS preparation mainly focuses on the simulation of fatty acid composition, the application of structured lipids and the addition of milk fat globule membrane (MFGM) supplements. This paper first reviews the composition and structure of HMF, and then the preparation development of structured lipids and MFGM supplements are summarized. Additionally, the evaluation and regulation of HMFSs in infant formula are also presented.

## 1. Introduction

Human milk is the most ideal source of nutrition in the early stages of human life. It not only provides comprehensive and balanced nutrition for infants but also greatly reduces their incidence of diarrhea, allergic diseases and infectious diseases and reduces infant mortality [1,2]. Human milk fat (HMF) is one of the most complex natural lipids, with a unique fatty acid composition and distribution. HMF provides about 50% of the energy needed for the growth and development of infants [3]. HMF also plays an important role in the absorption of vitamins and mineral nutrients, as well as in the early neural development and physiological functions of infants [4]. In recent years, the rate of breastfeeding has been decreasing for a variety of reasons. Therefore, infant formula has become the best substitute for human milk. With the in-depth study of HMF and infant growth needs, the production of human milk fat substitutes (HMFSs) has aimed to simulate HMF composition, which can narrow the gap between breast-fed infants and formula-fed infants.

Since the development of the first HMFS, many innovations have been implemented to improve the nutrient intake and absorption of infant formula. The composition and structure of HMFSs affect the bioavailability of lipids in infants, hence affecting their growth and development. This article reviews the structural composition and nutritional characteristics of HMF and the research progress of HMFS preparation, and the evaluation and regulation of HMFSs are also summarized.

## 2. Physiochemical Properties of HMF

**Figure 1:**
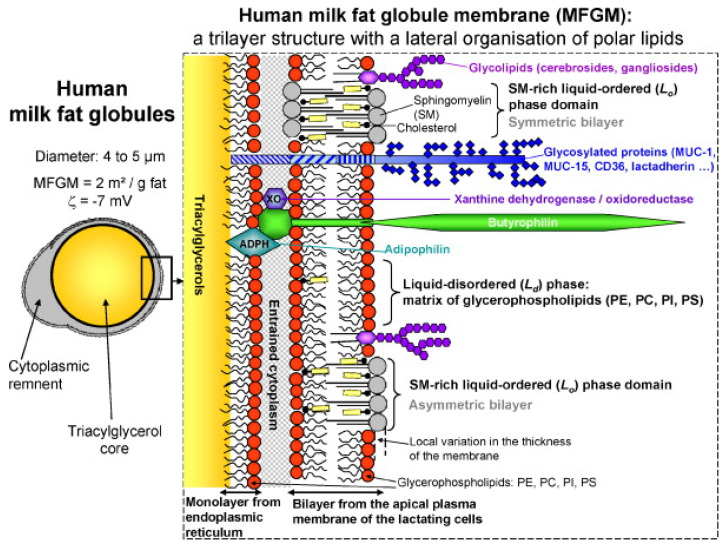
Structure of the milk fat globule and the membrane [11].

HMF is considered to be the reference or gold standard for the development of HMFSs [5,6,7]. Therefore, it is of great significance to study the composition of HMF. Generally speaking, HMF accounts for 3–5% of human milk, mainly containing triacylglycerols (TAGs, 98–99%), phospholipids (0.26–0.80%), sterols (0.25–0.34%, mainly cholesterol) and trace amounts of various minor components, including monoacylglycerols (MAGs), diacylglycerols (DAGs), fatty acids and other substances [8]. These components are packaged into milk fat lipid globules, with phospholipids forming the bulk of the milk fat globule membrane (MFGM) and TAGs found in the core. The MFGM mainly consists of a complex mixture of (glyco)proteins (20–60%), triacylglycerols, glycerophospholipids (33% of the MFGM), sphingolipids and cholesterol [9]. Other than lipids, the MFGM is also composed of glycosylated peptides, filaments, mucin and lactadherin (Figure 1). These globules usually range from 1 to 10 μm across, with an average diameter of 4 μm in mature milk [10].

The human MFGM has a complex architecture in which polar lipids (PLs) are the backbone. According to the model described by Lopez [11], PLs are organized in the MFGM as a trilayer with the polar head groups exposed to an aqueous environment (cytoplasm, aqueous phase of milk) and the hydrocarbon tails forming a hydrophobic area in the center of the bilayer or in contact with the TAG core. At least two lipid phases coexist in the outer bilayer of the human MFGM: (1) the liquid-disordered phase composed of glycerophospholipids and (2) the lateral segregation of sphingomyelin (SM) in liquid-ordered phase domains. The kinked structure of the unsaturated fatty acyl chains of glycerophospholipids results in a shorter molecular length compared to straight SM molecules that have long-chain saturated fatty acids (SFAs) and a sphingoid base. Hence, there is probably an interdigitation among the tails of SM molecules in the MFGM bilayer, and cholesterol can fill the voids between the heads of SM.

### 2.1. Fatty Acids and TAGs in HMF

TAGs, the most abundant component of HMF, contain three fatty acids. The different structures (chain length, number and position of double bonds) and positions along the glycerol backbone of fatty acids lead to the complex composition of HMF. HMF contains more than 200 types of fatty acid [12]. However, only several fatty acids dominate, and other fatty acids are present in very low concentrations. Fatty acids in HMF have unique distribution characteristics and commonly appear in specific positions. Interestingly, the distribution of fatty acids in glycerol influences their availability [13]. Table 1 summarizes the total and sn-2 fatty acid composition of HMF. According to the saturation degree, fatty acids can be divided into SFAs, monounsaturated fatty acids (MUFAs) and polyunsaturated fatty acids (PUFAs).

The amount of fatty acids in HMF is not constant and depends mainly on the lactation period, feeding stage, diet and health of the infant [17,18,19]. In most HMF, the dominating fatty acids are SFAs and MUFAs [20,21,22]. The sum amount of SFAs and MUFAs is between 83% and 86%, of which approximately half are SFAs [23]. Among SFAs, palmitic acid (C16:0) content is the highest, followed by stearic acid (C18:0), myristic acid (C14:0) and lauric acid (C12:0) [24,25,26]. The pH of stomach acid in infants is higher than that in adults, and their resistance to foodborne pathogens is weaker. Lauric acid has high antibacterial activity, which is beneficial for the resistance of infants and young children to pathogenic bacteria [27]. In addition, odd-numbered carbon chain fatty acids (C15:0 and C17:0) are not common in natural fats and oils but are widely found in HMF. Among MUFAs, oleic acid (C18:1) is the most prevalent, constituting around 90% of MUFAs in human milk, and it also accounts for a high percentage in the total pool of fatty acids [23]. The distributions of fatty acids on TAGs are similar in most human milk, with most SFAs attached to the sn-2 position. HMF contains about 20% palmitic acid, with the majority attached to the sn-2 position [28,29]. The major unsaturated fatty acids, including oleic acid and linoleic acid, occupy the sn-1,3 positions [30].

HMF contains about 10–22% PUFAs, with linoleic acid (C18:2) accounting for the highest content. According to the position of the last double bond, PUFAs are divided into two types: n-3 and n-6 [31]. In the n-3 family, the precursor in this group, α-linolenic acid (C18:3n-3; ALA), also dominates. PUFAs with a chain length of more than 20 carbons are called long-chain polyunsaturated fatty acids (LC-PUFAs). It is worth noting that among the fatty acids at the sn-2 position, LC-PUFAs, such as docosatetraenoic acid (C22:4), eicosapentaenoic acid (C22:5, EPA) and docosahexaenoic acid (C22:6, DHA), account for the highest sn-2 relative percentage (Table 1).

**Figure 2:**
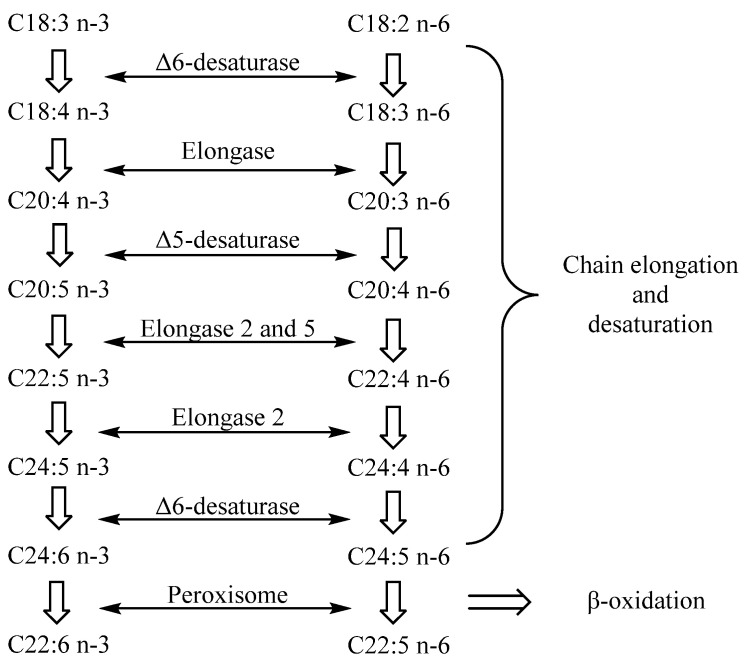
The flow chart of the synthesis of LC-PUFAs from linoleic acid and ALA through desaturation, carbon chain elongation and β-oxidation in vivo.

Arachidonic acid (C20:4, ARA) is the most common n-6 LC-PUFA in HMF, while common n-3 LC-PUFAs include DHA, docosapentaenoic acid (C20:5, DPA) and EPA. Several kinds of LC-PUFAs, such as DHA and ARA, exist at high concentrations in human central nervous tissue, especially in some specific brain regions and retinal cell membranes [32,33]. Their rapid accumulation in infants mainly occurs in the last three months of pregnancy and the first two years after birth, which is the period of rapid brain development in infants [34,35]. LC-PUFAs are important components of the neural cell membrane. It has been proved that LC-PUFAs can promote brain development by promoting neural stem cell differentiation, promoting synaptic formation and improving synaptic transmission mechanisms [36]. The postpartum period is the key period for the development of immune function in newborns. Research has shown that LC-PUFAs in HMF also play an important role in the improvement of the infant immune system [37,38]. As is shown in Figure 2, DHA and ARA can be synthesized in vivo through the action of elongase, desaturase and peroxisome on linoleic acid and ALA [39]. However, the enzyme system in infants is not well developed. Infants cannot synthesize enough LC-PUFAs to meet the requirements for growth, and thus, dietary supplements are prerequisite and vital.

### 2.2. Polar Lipids in HMF

In addition to neutral lipids, HMF also contains PLs, which are mainly distributed in the MFGM. PLs mainly include phosphatidylinositol (PI), phosphatidylcholine (PC), phosphatidylethanolamine (PE), phosphatidylserine (PS) and SM. Other PLs, such as lysophospholipids and plasmalogens, have also been found in human milk [40]. Despite their low content, PLs are important structural components due to their nutritional properties. Generally speaking, SM (range 7.96–16.49 mg/100 mL) is the most abundant PL in HMF, followed by PE (6.49–15.90 mg/100 mL), PC (3.63–8.09 mg/100 mL), PS (3.25–6.74 mg/100 mL) and PI (3.07–6.32 mg/100 mL) [11].

Table 2 summarizes the fatty acid composition of different types of PLs in HMF. Generally speaking, PC, PI and PS exhibit similar distributions of SFAs and unsaturated fatty acids (UFAs, around 50%). The UFA content in PE accounts for around 65%. However, SM presents a high SFA distribution of 70–80%, which helps to reduce liquidity and maintains the rigidity and structure of the MFGM [41]. Palmitic acid is the main fatty acid in PC, while stearic acid is the most abundant fatty acid in PE, PI and PS. Additionally, behenic acid (C22:0) is the main fatty acid in SM. ARA, EPA and DHA, which are vital to the growth and brain development of newborns, are mainly distributed in PE, PI and PS.

PLs derived from HMF have biological characteristics. As the precursors of intracellular messengers, PC and SM contents are considered to be very important for the growth and development of infants. For newborns and young children, PC and SM are the main sources of choline. About 17% of the total exogenous choline in newborns comes from these PLs [43]. Choline is an important nutrient that participates in a variety of biological processes, such as metabolism. It also exists in the membrane structure of the brain and nerve tissue [44]. Choline is the precursor of the neurotransmitter acetylcholine, which can regulate transduction signals and be a source of methyl groups in intermediate metabolism [45]. Choline is secreted from the maternal circulation into human milk and increases during lactation. The intact SM and its metabolites are important for the development of colon tumors, as well as the inflammatory process, cell function and growth [46,47,48].

Sphingolipids in the MFGM are believed to prevent globules from coalescing and to be critical for the formation and maintenance of the globular membrane structure [49,50]. The sphingolipids found in human milk include sphingomyelin and glycosphingolipids (glycolipids), and sphingomyelin (SM) is the most predominant species. Glycosphingolipids, which are quantitatively minor constituents of the MFGM, comprise cerebrosides (neutral glycosphingolipids containing uncharged sugars) and gangliosides (acidic glycosphingolipids containing sialic acid) [51]. The major ganglioside in HMF is monosialoganglioisde (GM_3_). Human milk GM_3_ is rich in saturated fatty acids, including palmitic acid (C16:0), stearic acid (C18:0), arachidic acid (C20:0), behenic acid (C22:0) and lignoceric acid (C24:0) [52]. Gangliosides provide sialic acid and have roles in immune protection in the infant by acting as prebiotic factors, as well as decoy receptors, or competing with pathogens for receptor sites on intestinal epithelial cells [53,54].

### 2.3. Digestion and Absorption of HMF

**Figure 3:**
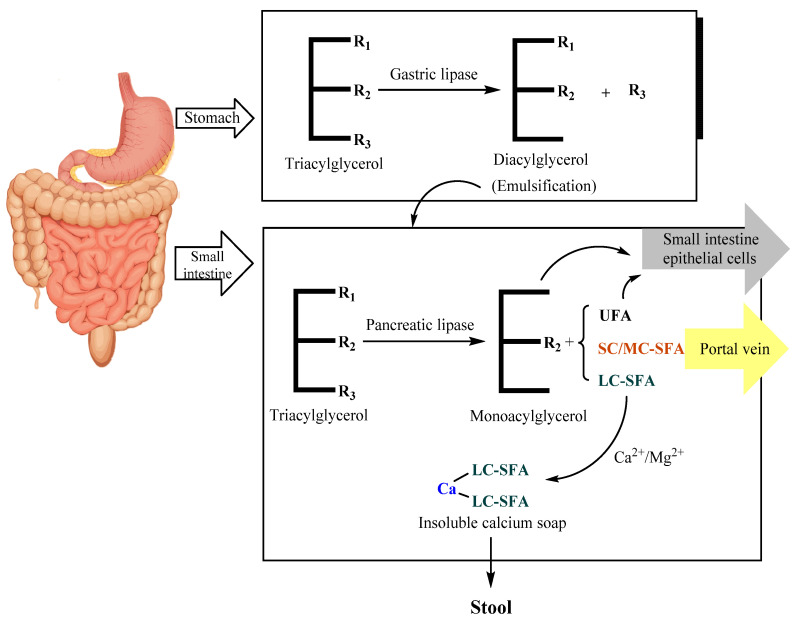
The digestion process of TAGs in infants. (R_1_–R_3_: random fatty acid; UFA: unsaturated fatty acid; SC/MC-SFA: short-/medium-chain saturated fatty acid; LC-SFA: long-chain saturated fatty acid).

As shown in Figure 3, TAGs in HMF undergo preliminary hydrolysis under the catalysis of gastric lipase. Gastric lipase is an sn-1,3-specific enzyme that preferentially hydrolyzes fatty acids at the sn-3 position and then generates free fatty acids (FFAs) and DAGs. Gastric lipase in infants can only hydrolyze 10–30% of TAGs [55]. DAGs have an emulsifying effect and help to further digest TAGs in the small intestine. Then, the initially digested HMF enters the small intestine and is hydrolyzed into sn-2 MAGs and FFAs under the catalysis of pancreatic lipase. Sn-2 MAGs are further transported to the brush border of the small intestine mucosa and absorbed by small intestinal epithelial cells [56].

The absorption of TAG hydrolysates is related to the composition and positional distribution of fatty acids [57]. The absorption of FFAs is affected by the chain length and degree of unsaturation [58]. SFAs with shorter carbon chains are transported to the liver for oxidation through the portal vein, while SFAs with longer carbon chains cannot pass through the portal vein [59,60]. In the environment of the small intestine in infants, free long-chain SFAs have a higher melting point than UFAs. They can easily combine with calcium and other metal ions to form insoluble soaps, causing the loss of fatty acids and calcium, as well as hard stools or even constipation [61]. Therefore, long-chain saturated fatty acids are absorbed more easily in the form of sn-2 MAGs [62].

As one of the most important fatty acids in HMF, palmitic acid is specifically distributed on TAGs, with more than 70% located at the sn-2 position [28,29]. Palmitic acid at the sn-2 position is more conducive to being absorbed and utilized [63,64]. Lipid digestion mainly relies on pancreatic lipase, which is sn-1,3-specific. Palmitic acid at the sn-1,3 position turns into FFAs after hydrolysis and binds with intestinal calcium and magnesium ions, generating insoluble fatty acid soap salt [65]. This can lead to low efficiency of fatty acid intake, loss of minerals and even stool sclerosis, constipation and other digestive diseases. In contrast, palmitic acid at the sn-2 position was proved to be absorbed much more efficiently [14]. Furthermore, palmitic acid at the sn-2 position plays an important role in improving the growth rate and the mineral density of bone, reducing infant crying and promoting the growth of intestinal probiotics [66,67].

At the same time, medium-chain fatty acids (MCFAs) are also important components in HMF, accounting for about 8–10%. MCFAs are easier to absorb than long-chain fatty acids (LCFAs). At present, manufacturers normally use medium-chain triacylglycerols (MCTs) to supplement MCFAs in infant formula. However, high levels of dietary MCTs may result in an undesirable increase in the level of circulating dicarboxylic acids, which can cause excessive ketone body poisoning [68]. It was found that the majority of MCFA-containing TAGs in HMF are medium- and long-chain triacylglycerols (MLCTs) rather than MCTs [69]. In addition, MCFAs can also limit the oxidation of PUFAs and LC-PUFAs and enhance the conversion of PUFAs to LC-PUFAs [70]. In MLCTs, MCFAs are released into the blood at a more stable rate of hydrolysis, avoiding excessive ketone body poisoning, and can also improve the absorption of LCFAs. MLCTs can also effectively reduce blood lipid and cholesterol levels, preventing and treating blood clots and other vascular diseases [71,72].

## 3. Production of HMFSs

### 3.1. Blending

Blending refers to the process of simply blending different animal fats, vegetable oils or microbial oils to prepare HMFSs. It has the advantages of simple operation and lack of by-products, and it can be used in large-scale commercial production. The development of HMFSs began in the 19th century, with the goal of mimicking the lipid content of HMF. Most infant foods of the first generation, represented by the first commercial infant formula developed by Leibig, had high total calories from protein and low values from fats, among which bovine milk fat (BMF) was applied as HMFSs [73,74].

At the beginning of the 20th century, the difference in fatty acid composition between BMF and HMF was clearly recognized. Hence, the composition of infant formula underwent “humanization” transformation. Gerstenberger developed blends of tallow, tallow oil, coconut and cocoa butter to mimic HMF. Based on the chemical analysis available at the time, it was determined that this mixture of fats was nearly identical to HMF in terms of average molecular weight, saturation and proportion of fatty acid [75]. Cod liver oil was used in some formulas decades before the important role of DHA in infant development was discovered [76].

At present, most commercial infant formulas still contain HMFSs prepared by pure blending. The main types are as follows: 1. A mixture of single vegetable oil and milk fat; 2. A mixture of various vegetable oils and milk fats; 3. A mixture of vegetable oils. Generally, the combination of commercial vegetable oils (coconut oil, palm oil, high oleic safflower oil or sunflower oil, soybean and rapeseed oil) is a good source of SFAs (C8:0–C18:0). Simple mixtures of unsaturated vegetable oils (e.g., corn or soy oil) and fats that contain a predominance of lauric acid or shorter saturated fatty acids (e.g., coconut oil) are common fat blends in some formulas. Complex mixtures of oils that contain modest levels of long-chain SFAs can be used in developing formulas with fatty acid profiles closer to that of human milk [57]. MCTs, predominantly containing caprylic acid (C8:0) and capric acid (C10:0), have been added to some preterm formulas to maximize fatty acid absorption in preterm infants [77].

Approximately 70% of palmitic acid in human milk is in the sn-2 position, whereas this fatty acid is primarily in the sn-1 and sn-3 positions in most animal and vegetable fats. Lard contains approximately 80% of its long-chain SFAs in the sn-2 position, which is similar to the SFA content in HMF. Therefore, several studies have used lard-based formulas and proved their efficient fat absorption [63,64]. However, there are numerous cultural and religious prohibitions against using lard. In the late 20th century, multiple studies used blends of palm olein and other vegetable oils in different ratios to achieve proportions of palmitic and oleic acid that were similar to those in HMF [78,79,80,81]. Through comparison, they proved that infant formulas with palm olein at levels similar to HMF lower the absorption of calcium and/or fat with increased stool hardness and lower bone mass.

PUFAs (especially DHA and ARA) are supplemented from docosahexaenoic acid single cell oil (DHASCO), arachidonic acid single cell oil (ARASCO) and marine oil [82,83]. The fatty acid compositions of commonly used oils are shown in Table 3 and Table 4. Fish oil generally contains DHA, EPA and small amounts of ARA and DPA, and their composition varies depending on diet, location, season and physiological conditions (such as species age and gender) [84]. Recently, it was found that the reason for the high content of LC-PUFAs in marine fish oil is that marine fish feed on plankton consuming microalgae, which have the ability to synthesize and accumulate a large number of LC-PUFAs [85]. In addition, some microorganisms, such as bacteria and fungi, have also been proved to have the ability to synthesize LC-PUFAs. A study confirmed that LC-PUFAs derived from single cells have no adverse effects on the growth and overall health of infants [86]. At the same time, it is necessary to ensure reasonable proportions of PUFAs during blending. An imbalance between n-3 and n-6 LC-PUFAs will affect the development of language function and the growth and development of children [87]. Additionally, different LC-PUFAs should be reasonably added during the preparation of HMFSs. An imbalance of dietary DHA and ARA may have negative impacts on cognitive development [88,89].

Some studies have proved that HMFSs in commercial milk powder can imitate HMF at the fatty acid level [100]. However, the blending method can only change the fatty acid composition and content of HMFS. The distribution of fatty acids in traditional HMFSs is far from that of HMF, and thus, it cannot meet all of the nutritional needs of infants. Therefore, the preparation of HMFSs is not limited to unmodified oil but has started to include structured lipids (SLs) as raw materials [101,102].

### 3.2. Preparation of SLs

In 1968, Wyeth published the results of an animal study showing that a blend with a high proportion of sn-2 palmitate oil was well absorbed and was better than other blends, including butterfat, lard and vegetable oils [103]. These results offered an explanation for the superior absorption of HMF that could not be explained by fatty acid composition alone. The importance of the positional distribution of fatty acids has been highly valued. Hence, the preparation of SLs has attracted much attention. The main methods of SL preparation include the chemical method and the enzymatic method. Although chemical synthesis is an important process in the industry, it has many disadvantages in general, such as high reaction temperature, weak specificity, many reaction by-products, poor product flavor, high requirements of equipment, etc. Therefore, many researchers have focused on enzymatic synthesis. The enzymatic synthesis method has many advantages, such as mild reaction conditions, simple separation and purification steps, and strong product specificity, which can synthesize HMFSs with special structures to meet the needs of infant growth and nutrition [104].

#### 3.2.1. Enzymatic Acidolysis

**Figure 4:**
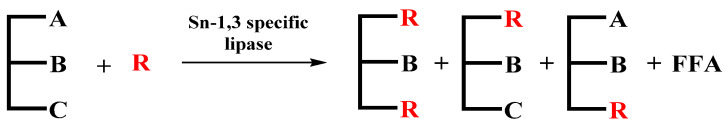
Acidolysis process route of structured lipids (A–C: original fatty acids; R: random fatty acid).

Acidolysis is the most commonly used method for the preparation of SLs [105]. As is shown in Figure 4, enzymatic acidolysis is a method in which TAGs exchange acyl groups with fatty acids under the catalysis of lipase. During the acidolysis process, fatty acids at the sn-2 position easily migrate to the sn-1,3 positions in the intermediate by-product (DAGs), which ultimately leads to a decrease in the purity of the target product [106]. This method has the advantages of simple operation, fewer kinds of products and easy separation, which makes it the most commonly used synthetic method at present [107].

##### Preparation of sn-2 Palmitate SLs through Acidolysis

The acidolysis reaction is mainly applied in the preparation of sn-2 palmitate SLs. Most studies have used palmitic acid–rich sources, among which the most common one is tripalmitin [107]. Ilyasoglu et al. [108] synthesized HMFSs by Lipozyme^®^ RM IM (*Rhizomucor miehei* lipase)-catalyzed acidolysis among tripalmitin, hazelnut oil fatty acids and Neobee^®^ fatty acids. The SLs contained caprylic acid (12.8 g/100 g), capric acid (10.6 g/100 g) and palmitic acid (30 g/100 g) [106].

On the other hand, other researchers have used animal fat as the raw material to produce HMFSs. Lard is a rich renewable resource in China and contains 29.5% palmitic acid and 74% at the sn-2 position, which is similar to human milk [109]. However, this renewable resource cannot be effectively used because lard has a high melting point and mainly contains LCTs, which affects its physical, chemical and physiological properties and limits its application in the food industry. In the study of Yang et al. [110], lard and fatty acids from soybean were esterified in a solvent-free system at both the laboratory scale and large scale, producing HMFSs containing 71.1% sn-2 palmitic acid and 44.4% sn-1,3 oleic acid. Zhao et al. [111] synthesized SLs that incorporated 34.2 mol% capric acid through acidolysis between lard and capric acid in a hexane system. Nielsen et al. [112] produced HMFS through acidolysis of lard and fatty acid from soybean oil. The content of sn-2 palmitic acid in the product accounted for 71.9%, and the content of linoleic acid and linolenic acid increased from 9.2% and 0.8% to 23.8% and 2.3%, respectively. Basa catfish oil is a newly discovered natural oil whose fatty acid composition and distribution are similar to those of HMF. Zou et al. [113] prepared SLs containing 58.43% sn-2 palmitic acid and 80.16% sn-1,3 oleic acid through a two-step process from basa catfish oil. The process includes fractionation to enrich TAG fractions with a high content of sn-2 palmitic acid and Lipozyme^®^ RM IM-catalyzed acidolysis of the fractionated products with FFAs from high-oleic-acid sunflower oil to increase the content of 1,3-oleoyl-2-palmitoylglycerol (OPO). However, consumers may be reluctant to use animal fat to feed infants. Therefore, in recent years, there have been studies using algal oil rich in palmitic acid [114].

In addition to natural lipids, some studies have used a two-step acidolysis method to prepare SLs in order to increase the content of sn-2 palmitic acid. Jiménez et al. [115,116] first obtained FFAs rich in palmitic acid through saponification and fractionation and then used an acidolysis reaction between FFAs and palm stearin to prepare SLs with an sn-2 palmitic acid content of up to 78.5%. These palmitic acid–rich TAGs can be used to obtain structured TAGs rich in palmitic acid at the sn-2 position and oleic acid at sn-1,3 positions. Esteban et al. [117] prepared palmitic acid–enriched acylglycerols with palmitic acid and palm stearin through two-step acidolysis and then used oleic acid–enriched FFAs for further enzymatic acidolysis. In the solvent and solvent-free systems, SLs with up to 67.2% oleic acid at sn-1,3 positions and 67.8% palmitic acid at the sn-2 position were obtained under the applied conditions.

##### Preparation of SLs Rich in LC-PUFAs through Acidolysis

LC-PUFAs are important components of HMFSs due to their vital influence on growth and development. SLs rich in LC-PUFAs are often prepared through the acidolysis process. Sahin et al. [118] prepared fatty acid and fatty acid ethyl ester by hydrolysis and alcoholysis of DHASCO and ARASCO, respectively. The FAEEs were used as acyl donors for acidolysis and transesterification with tripalmitin. After purification, the content of sn-2 palmitic acid in the product reached 48.53%, and the contents of DHA and ARA were 10.75% and 17.69%, respectively. Through a similar process, Li et al. [119] enriched refined olive oil with palmitic acid and DHA through acidolysis. In the product, palmitic acid was incorporated into the TAGs of refined olive oil at 55.79 mol%. Meanwhile, the incorporated palmitic acid at the sn-2 position and total DHA were found to be up to 33.63 mol% and 3.54 mol%, respectively. Pande et al. [120] used tripalmitin, extra virgin olive oil FFAs and DHASCO FFAs as the substrates of acidolysis. In their product, sn-2 palmitic acid accounted for 60 mol%, and the oleic acid content in sn-1,3 positions was predominant, while the DHA content accounted for 5.89–7.54% in different SLs. In the study of Nagachinta and Akoh [121], DHA and γ-linolenic were incorporated into SL products to improve their nutritional value while maximizing the content of palmitic acid at the sn-2 position. The SL product contained 35.11% palmitic acid at the sn-2 position, with 3.75% DHA and 5.03% γ-linoleic acid. Nagachinta and Akoh [122] synthesized SLs containing 56.9 mol% sn-1,3 ARA and 28.7 mol% sn-2 palmitic acid through enzymatic acidolysis between ARA and tripalmitin.

Though SLs rich in sn-1,3 LC-PUFAs have a positive impact on lipid metabolism [123], considering the physiological function and stability of the sn-2 position LC-PUFAs, recent studies have explored the preparation of SLs rich in LC-PUFAs at the sn-2 position. For example, Zou et al. [124] and Naranjo et al. [125] prepared SLs containing 87.45 and 54.4 mol% sn-2 LC-PUFAs through acidolysis between microbial oil and MCFAs, respectively.

#### 3.2.2. Enzymatic Interesterification

**Figure 5:**
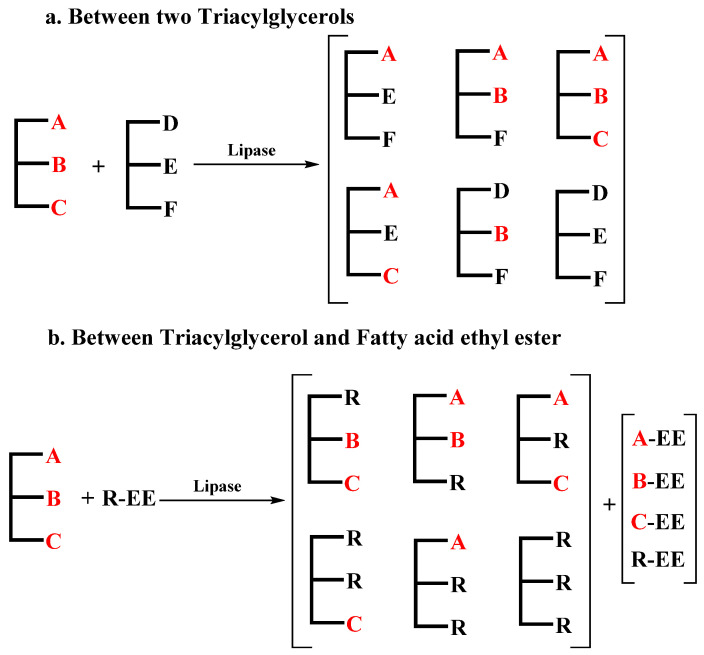
Process route of enzymatic interesterifications between two triacylglycerols (**a**) and between triacylglycerol and fatty ethyel ester (**b**) (A–F: fatty acid; R: random fatty acid; A-C/R-EE: fatty acid ethyl ester).

As is shown in Figure 5, interesterification mainly refers to the enzymatic reaction between different TAGs or between TAGs and fatty acid ethyl ester. Due to its low specificity, low synthetic efficiency and difficult separation of reaction products, interesterification, especially reactions between TAGs, is not widely used. Enzymatic interesterification is a directional process catalyzed by lipase. Depending on enzyme regioselectivity, enzymatic interesterification can be classified as random or specific. Intermediate specificity can also be obtained simply by adjusting the residence time of the enzymatic process [125]. Due to the high selectivity of lipase, enzymatic interesterification only produces a few unwanted by-products, leading to fewer post-treatment products. Enzymatic interesterification is carried out under mild operating conditions, resulting in a lower level of PUFA degradation.

Maduko and Park [126] modified the structure of TAGs in a vegetable oil mixture under the catalysis of Lipozyme^®^ RM IM through interesterification between the vegetable oil mixture and tripalmitin. These authors studied the incorporation of palmitic acid, oleic acid and linoleic acid at the sn-2 position and obtained a product with a similar fatty acid profile to that of HMF. In the product, MLCTs with palmitic acid at the sn-2 position accounted for 64–66%. Korma et al. [127] used the interesterification of ARASCO and MCTs in a solvent-free system to prepare SLs with an MLCT content of 53.75%. In the research of Ghosh et al. [128], the content of palmitic acid in TAGs was increased by fractionated palm stearin. The product of interesterification between palm stearin fractionate and fish oil contained 75.98% palmitic acid at the sn-2 position, as well as 0.27% ARA, 3.43% EPA and 4.25% DHA. Bektas et al. [129] used ethyl oleate as the acyl donor, which greatly increased the acyl group incorporation rate, and the sn-1,3 oleic acid content reached 64.9%, while its sn-2 palmitic acid content remained at around 80.6%. Rodriguez and Akoh [130] synthesized sn-2 palmitic acid–rich SLs through the interesterification reaction of amaranth oil, DHASCO and ethyl palmitate and then prepared HMFSs containing 33.9% palmitic acid and 1.9% DHA.

#### 3.2.3. Enzymatic Esterification

**Figure 6:**
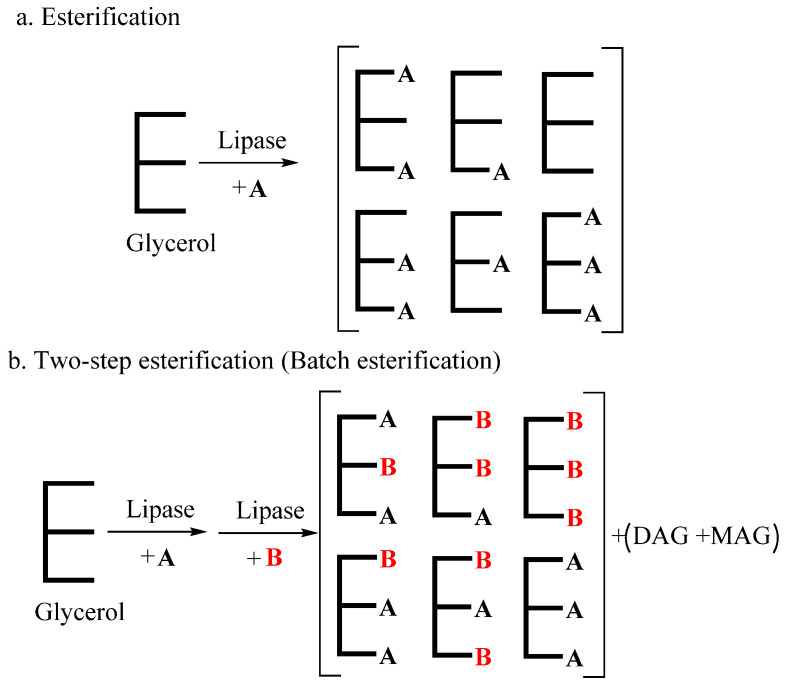
Process routes of **one-step (a) and two-step (b)** enzymatic esterification (A–B: fatty acid).

There are only a few studies on esterification in SL production. As is shown in Figure 6, esterification is a reaction between fatty acids and glycerol under the catalysis of lipase. It has the advantage of enabling direct usage of by-products containing FFAs and glycerol [131]. Since industrial quantities of FFAs can be obtained from other industrial production processes, esterification can be used to produce SLs for food applications. Currently, esterification reactions catalyzed by lipases are mainly used in the synthesis of DGs, such as 1,3-diolein [132]. In addition, the common method of producing SLs through an esterification-based process involves MLCTs. Yang et al. [133] implemented a one-step esterification process in the production of MLCTs based on caprylic acid, capric acid and oleic acid with glycerol, resulting in 72.19% MLCT yield. However, there are many other glyceride by-products of one-step esterification, such as DAGs and MAGs, and the fatty acids can only be randomly arranged on the glycerol backbone. Nagao et al. [134] used esterification for the synthesis of SL-containing DHA. The product contained 51.7 mol% DHA at sn-1,3 positions, while the sn-2 DHA content was 17.3 mol%. This can be attributed to the large steric hindrance of LC-PUFAs and the higher catalytic activity of lipase for other fatty acids.

Two-step esterification has been used to synthesize DAGs and TAGs step by step, which includes adding FFAs in batches. Agapay et al. [135] used two-step esterification for the preparation of OPO, with oleic acid, palmitic acid and glycerol as reaction substrates. The OPO content in the prepared product was 34.98–39.55 wt%, which matched that of acidolysis products in several studies. In the study of Medina et al. [136], a 1,3-dicapryloyl-2-PUFA glycerol product containing about 90 wt% TAGs was synthesized using concentrated PUFAs from sardine discards through two-step esterification. The two-step process usually utilizes different lipases for each step and involves the intermediate purification of precursors between the steps.

#### 3.2.4. Combination of Alcoholysis and Reesterification

**Figure 7:**
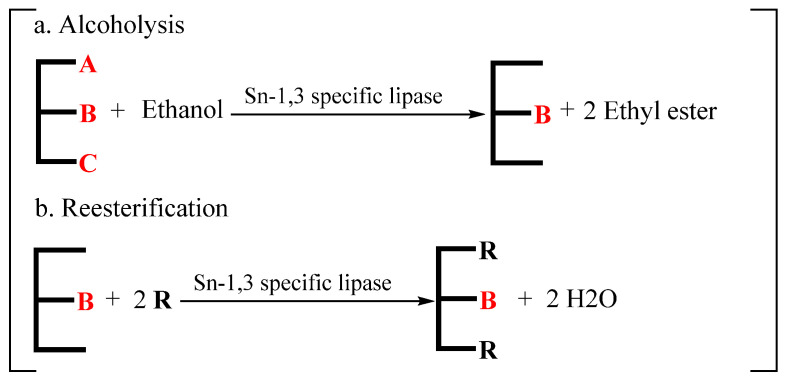
Process route of enzymatic alcoholysis (**a**) and reesterification (**b**) (A–C, R: fatty acid).

In order to obtain SLs with high purity, the combination of alcoholysis and reesterification has been conducted by a large number of researchers. As is shown in Figure 7, the first step is alcoholysis between TAGs and ethanol under the catalysis of sn-1,3-specific lipase to produce sn-2 MAGs, which are not stable and are easy to convert to sn-1,3 MAGs. Then, the second step is acidolysis between MAGs and fatty acids.

Since the product of this reaction retains fatty acids at the sn-2 position to the greatest extent, this method is often used to prepare SLs rich in specific fatty acids at the sn-2 position, such as sn-2 palmitate SL and SLs rich in sn-2 LC-PUFAs. Schmid et al. [137] and Pfeffer et al. [138] synthesized OPO through a two-step enzymatic process catalyzed by sn-1,3-specific lipases, which included alcoholysis of tripalmitic acid with ethanol to produce sn-2 palmitate MAG and then esterification between sn-2 MAGs and oleic acid. After purification, the obtained products contained 96% sn-2 palmitic acid, 90% sn-1,3 oleic acid and 95% OPO purity. Irimescu et al. [139] selected tridocosahexaenoylglycerol and ethylcaprylate as raw materials and obtained products (1,3-dicapryloyl-2-docosahexaenoyl glycerol (CDC), 85.4% yield) with regioisomeric purity reaching 100% after a two-step enzymatic process.

Table 5 summarizes the main studies on the preparation of SLs by enzymatic methods. Although the purity of SLs obtained by a two-step enzymatic method is very high, the complicated steps make this method less attractive.

#### 3.2.5. Fermentation with Microorganisms

All of the enzymatic methods require expensive lipases, which limits their industrial feasibility [154]. Although immobilized lipase can be recovered, a decrease in catalytic activity is inevitable [155]. Therefore, more green and sustainable methods for the synthesis of HMFSs need to be developed. Microorganisms may be a good choice for OPO production. Microbial oils have many advantages and have been identified as a promising alternative to vegetable oils and animal fats because of their season-independent production and adjustable composition [156].

**Figure 8:**
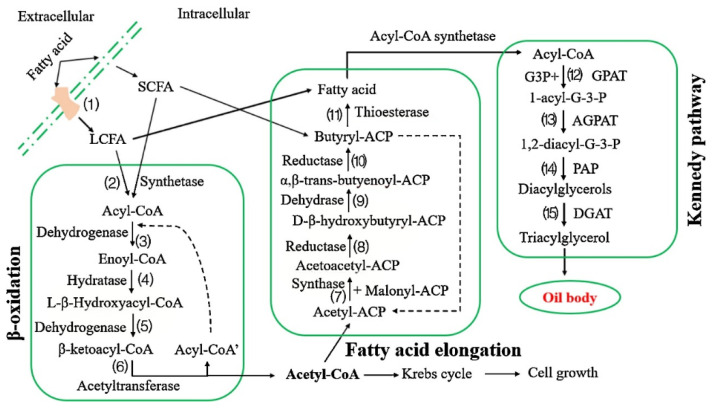
Scheme of fatty acid metabolism in R. opacus (LCFA, long-chain fatty acid; SCFA, short-chain fatty acid; G3P, glycerol-3-phosphate; GPAT, glycerol-3-phosphate acyltransferase; 1-acyl-G-3-P, 1-acyl-sn-glycerol-3-phosphate; AGPAT, 1-acyl-sn-glycerol-3-phosphate acyltransferase; 1,2-diacyl-G-3-P, 1,2-diacyl-sn-glycerol-3-phosphate; PAP, acylphosphatate; DGAT, diacylglycerol acyltransferase) [152].

The potential for producing HMFSs by fermentation using *Rhodococcus opacus* was explored in the study of Zhang et al. [152]. Transcriptome analysis showed that β-oxidation, fatty acid elongation and Kennedy pathways exist in *R. opacus*. The fatty acid supplied as a carbon source can enter the Kennedy pathway directly or through a de novo biosynthetic pathway according to the chain length. As is shown in Figure 8, the results indicated that different compounds with chain lengths from 12 to 18, used as a carbon source, could be incorporated into TAGs directly. PUFAs, including ARA, EPA and DHA, could enter the Kennedy pathway directly and be involved in the biosynthesis of TAGs. Specifically, if the chain length was less than 12, the carbon source entered the fatty acid elongation pathway and formed palmitic acid or oleic acid. When oleic acid, palmitic acid and linoleic acid were supplied as substrate fats, 1-oleoyl-2-palmityl-3-linoleoyl glycerol (OPL) was formed [152,153].

In the study of Zhang et al. [153], chemically interesterified fat or a mixture of ethyl oleate/ethyl palmitate 2:1 (*w*/*w*) was used as a starting material. After fermentation with *R. opacus*, OPO (47.1%), OPL (13.9%), PPO (13.1%) and PPoO (16:0–16:1–18:1) (10.3%) were the most abundant TAG species. Its feasibility of large-scale production has also been proven. The main component of the oil produced by *R. opacus* is TAG, which shows that it can be used as edible oil after proper refining. Furthermore, compared with the enzymatic method, no acyl group migration was detected during the fermentation process [152].

### 3.3. Purification and Treatment of SLs

Regardless of whether HMFS is prepared by chemical or enzymatic catalysis, the reaction products contain by-products, such as FFAs and DAGs. Removing by-products by effective methods improves not only the quality but also the nutritive value of HMFSs. Purification methods mainly include physical methods and chemical methods [157,158]. Chemical purification methods, such as urea inclusion and solvent crystallization, consume a large amount of solvent that is difficult to fully recover, resulting in high cost and solvent residue [159,160]. Therefore, chemical methods are often used in laboratories. Physical methods include supercritical carbon dioxide extraction, distillation, molecular distillation, etc. Molecular distillation is a safe, green and rapid purification method. It has the advantages of a high degree of separation, short material residence time and low distillation temperature. It is suitable for the separation of substances with a high boiling point and high heat sensitivity [115,150].

From a stability point of view, PUFAs are highly unstable and tend to oxidize. PUFAs have at least two double bonds, which are easily attacked by active agents. The resulting PUFA hydroperoxides may lead to secondary reactions, a bad smell and bioactivity loss [161]. Therefore, extra protection for PUFA products is necessary. LC-PUFAs in infant formula are mostly added through the microcapsule process. In the microcapsule process, natural or synthetic polymer materials are used as capsule materials, and SLs are embedded in wall materials by chemical, physical or physicochemical methods [162]. This process can protect LC-PUFA-containing SLs, mask the bad flavor and release SLs at a controllable rate under specific conditions. There are many methods to achieve microencapsulation, such as spray condensation, freeze-drying, etc. [163,164]. Among them, spray drying is the most widely used method in the food industry [165]. The principle is that the emulsion is atomized, and the solvent is rapidly evaporated by high-temperature airflow. Finally, the wall material forms a membrane structure on the core surface. The process has several advantages, including low cost, high controllability of process conditions, uniform particles and good solubility of the product [166]. For the microencapsulation of LC-PUFAs, the wall material should be food-grade ingredients and be suitable for infants, among which natural ingredients are preferred. Milk protein, a milk protein–carbohydrate mixture and their Maillard products are good wall materials [167]. In order to prevent the oxidation of LC-PUFAs during microencapsulation, some natural antioxidant components, such as tocopherol, can be added. This can further improve the stability of LC-PUFAs in infant formula [168].

### 3.4. Preparation of MFGM

**Figure 9:**
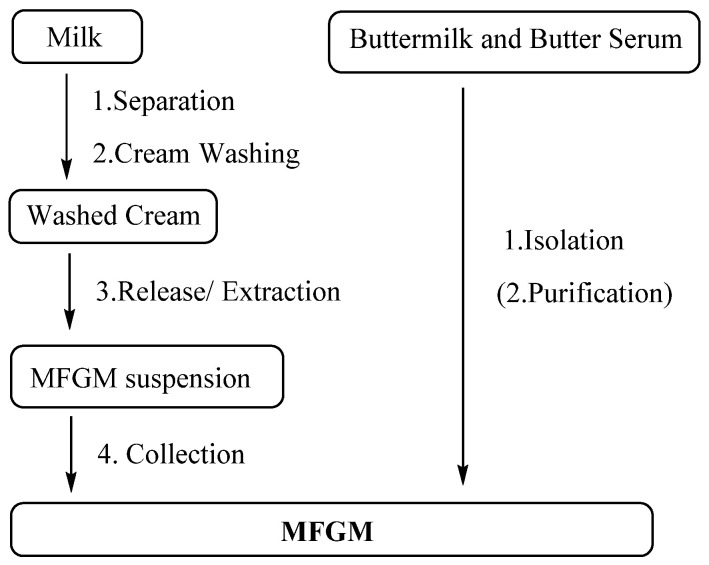
Process route of MFGM production.

PLs have been added to infant formula as biologically active elements due to their potential benefits for the optimal growth and health of infants. MFGM supplements and polar lipid-rich ingredients are mainly extracted from bovine milk. As is shown in Figure 9, the separation method of the MFGM can generally be divided into four steps: fat globule separation, cream washing, release of the MFGM from the globules and collection of MFGM materials [169]. Membrane proteins are highly vulnerable and easily lost during isolation, especially loosely bound proteins; around 16% of MFGM proteins were lost during production. Luckily, only 4% of phospholipids were lost [170]. Washing also causes losses of tocopherol, which is an antioxidant in the MFGM [171]. Bovine serum albumin could still be detected in the MFGM product despite successive washings [172].

The preparation of the MFGM from fresh industrial liquid butter serum or buttermilk mainly requires isolation, including micro- and ultrafiltration, and renneting coagulation. Citrate has also been used to dissociate casein micelles [173,174,175]. More phospholipids can be separated by further purification, including supercritical fluid extraction and solvent extraction.

### 3.5. Commercial HMFS Products

Due to the positive effect on infant health, the most popular commercial HMFS product is sn-2 palmitate SL [176]. A variety of sn-2 palmitate SL products on the market and their compositions are listed in Table 6. As the first commercialized sn-2 palmitate SL product developed by IOI Loders Croklaan, Betapol^®^ has been used in infant formulas since 1995 [177]. The main components in Betapol^®^ are OPO and 1-oleoyl-2-palmitoyl-3-linoleoylglycerol/1-linoleyl-2-palmitoyl-3-oleoylglycerol (OPL/LPO). In addition, Advanced Lipids developed InFat^®^, in which 70–75% of palmitic acid is distributed at the sn-2 position [178,179]. In order to meet the needs of the Chinese market, these companies have developed OPO products that mimic the composition of Chinese HMF, such as Betapol^®^ Select and InFat^®^ Plus. In addition, Wilmar developed Milkopas^®^ 9320, in which over 62% of palmitic acid is distributed at the sn-2 position, and the content of OPO is over 53%. The contents of sn-2 palmitic acid and OPO are higher than the Chinese national standard and much closer to the contents in HMF. As one step closer to the goal of “humanization”, Milkopas^®^ has taken one-third of the current market share. As an LC-PUFA product, Nature’s One developed Baby’s Only^®^ Essentials DHA & ARA Fatty Acid, which is naturally derived from egg phospholipids using an aqueous process [107].

Some MFGM supplement products are already on the market and have been added to some infant formulas in the Chinese market. For instance, Lacprodan^®^ MFGM-10 by Arla Foods Ingredients Group is a protein-rich MFGM supplement derived from bovine milk, containing 73% protein, 8% MFGM protein, 7% phospholipid, 2% sialic acid, 5% IgG, 0.2% lactoferrin and 0.2% ganglioside [180], and BSC by Fonterra Co-operative Group contains 52.30 g protein, 36.20 g lipid with 13.67 g phospholipid and 0.63 g ganglioside, 6.60 g lactose and 5.20 g minerals per 100 g. The MFGM from Fonterra consists of 647 mg/L phospholipids, including 57 mg/L PI, 80 mg/L PS, 173 mg/L PC and 141 mg/L SM. In addition, Inpulse from Büllinger SA has a nine-fold higher concentration of phospholipids compared to that found in Lacprodan^®^ PL-20 (Arla) and contains 16% phospholipid (SM, gangliosides) and about 55% proteins [181].

## 4. Fat-Related Regulations and Guidelines for Infant Formula

In 1938, the U.S. Federal Food, Drug, and Cosmetic Act (FFDCA) contained the first reference to foods for special dietary purposes, including infant formulas [182]. The act helped standardize the branding of food, setting standards for identity, quality and fill volume or weight [183]. The Infant Formula Act of 1980 clarified the US Food and Drug Administration (FDA)’s authority to establish minimum nutrient requirements and to establish quality control procedures [184]. It also established maximum permissible levels of protein, fat, sodium, potassium, chloride and vitamins A and D for the first time.

At present, the current legislation on infant formula is set by different authorities, including the Codex Alimentarius Commission (CAC), FDA, the European Commission (EC) and the National Health Commission of the People’s Republic of China (NHC) [107]. The maximum and the minimum contents legislated for infant formula lipids are summarized in Table 7. As the compulsory composition, total fat and linoleic acid content within 0.3–3.0 g/100 kcal are required by all authorities. ALA is required to be more than 50 mg/100 kcal by CAC, EC and NHC. Trans fatty acids should be less than 3% of total fatty acid, and erucic acid should be less than 1% of total fatty acid. Phospholipids should not exceed 2 g/L, according to CAC and EC. LC-PUFAs are permitted as facultative additions by CAC, EC and NHC [185,186].

DHA is now recommended as a required constituent. In 2005, the Nutrition Committee of the European Society of Pediatric Gastroenterology, Hepatology and Nutrition (ESPGHAN) proposed a global standard for the composition of infant formula. The standard includes 7 and 50 mg choline/100 kcal as the recommended minimum and maximum values, respectively [187]. Requirements for infant formula in the USA indicate a minimum of 7 mg/100 kcal for infant formula in general [188,189,190,191]. The Department of National Health and Welfare Canada established that the choline content of formula should be no less than 12 mg/100 kcal. The Food and Nutrition Board [192] has set an adequate intake of choline of 125 mg/day for infants from 0 to 6 months of age.

## 5. Evaluation of HMFSs

With the popularization of infant formula, a wide variety of commercial products for infant formula with varying quality emerged. Meanwhile, more and more studies have been working on the analysis and evaluation of the composition of HMFS [92,193,194]. In order to further evaluate the quality and potential application prospects of synthetic HMFSs, it is also vital to evaluate the quality of refined HMFS products. The characterization of the conventional physical and chemical indicators of the product include saponification value, acid value, peroxide value and oxidation stability [195,196,197].

Due to its advantage in the absorption of fatty acids, minerals and vitamins and its ability to reduce constipation, HMF is regarded as the “golden standard” and provides a direction for the development of HMFSs [5,6,7]. Therefore, the evaluation of the similarity between HMFSs and HMF is also indispensable. However, the wide variety and the complex positional distribution of fatty acids in HMF have made the evaluation difficult. Although composition analysis can clarify the difference in compositions between HMFSs and HMF, it cannot intuitively and quantitatively evaluate the similarity [8,198]. In order to evaluate the similarity of HMFSs, it is necessary to establish scoring models. A scoring model will promote the development of the infant formula industry towards standardization. At present, there are only a few reports on establishing similarity evaluation methods of HMFSs.

Wang proposed a two-dimensional scoring model based on the principle of “deduction”; that is, the total and sn-3 fatty acids in HMF are used as a reference standard [8]. The greater the deviation between the corresponding content of HMFSs and the reference value of HMF, the lower the similarity score. The effectiveness of the evaluation model was verified by evaluating 15 different fats in this study, of which 5 samples were infant formula milk powder. The results showed that lipids in infant formula had a high similarity to HMF in terms of fatty acid composition but a generally low similarity in terms of the distribution of fatty acids [8].

In addition, Zou et al. [198] proposed a four-dimensional scoring model based on the main fatty acids, the relative content of sn-2 fatty acids, PUFAs and the main TAGs of HMF as reference standards. The authors evaluated three infant formula samples using this model, and the results showed that the similarity of the main fatty acids was relatively high (89.4–98.4), but the similarity of the relative content of sn-2 fatty acids and PUFAs was generally low, with values of 40.2–58.6 and 28.9–67.2, respectively [198].

In a recent study, Kloek et al. [199] extended the model of Wang et al. [8] and included sn-1,3 fatty acid composition, and fatty acids at sn-1,3 positions were weighted two-thirds in the average index. Fatty acids positioned at sn-1,3 could be linked to fatty acid soap formation and calcium bioavailability in infants. Therefore, sn-1,3 fatty acids should be involved in the evaluation of HMFSs. According to their results, a higher average SI was observed in bovine milk fat–containing infant formulas compared with palm oil–containing or palm oil–free infant formulas [199].

The first two classic scoring models have been applied for evaluations by researchers [200,201,202,203,204]. Zou et al. [205] evaluated the potential of mammalian milk fats for HMFSs based on the total and sn-2 fatty acid, TAG, phospholipid and phospholipid fatty acid compositions. According to the results, all of the milk fats tested had high degrees of similarity to HMF in total fatty acid composition. However, the degrees of similarity in other chemical aspects were low, indicating that these milk fats did not meet the requirements for HMFSs [205]. Zou et al. [200] also used a scoring model to evaluate the similarity of HMFS synthesized by an enzymatic process, in which the relative content of sn-2 fatty acids and the similarity of TAGs were improved. In addition, the lipids in commercial infant formulas were evaluated by the models. The results indicated that the scores of vegetable oil–based formulas were in the range of 0–49.5, whereas the scores of formulas based on cow and goat milk were in the ranges of 5.7–53.2 and 26.4–52.7, respectively. All commercial HMFSs had scores below 60. The overall score of 180 commercial HMFSs was 38.4 [100,204,206].

## 6. Conclusions

Human milk is the first-choice food for infants, and HMF plays an important role in the development of infants. In the past few years, great progress has been made in HMFS preparation. At present, HMFSs can effectively simulate the fatty acid composition of HMF, and LC-PUFAs are supplied through the addition of deep-sea fish oil or microalgae oil to meet the needs of infants. With the in-depth study of HMF, its composition and structure are well known. More attention is paid to the more in-depth simulation of HMFSs at the TAG level. Thus, the first challenge in the development of HMFSs is the evaluation of HMFSs. The scoring model should be further studied at the TAG level or even in the aspect of the milk fat globule. This could further inform the research and production of HFMSs. At present, the synthesis of SLs, such as sn-2 palmitate SL and LC-PUFA-enriched SLs, is mainly synthesized by acidolysis and the combination of alcoholysis and esterification. However, the high cost of enzymatic synthesis limits the application of SLs. The production cost should be further reduced through research for more suitable food-grade lipase, immobilization materials, substrates and process methods to realize industrial production of SLs. In addition, more attention should be paid to fermentation synthesis and the formulation of relevant laws and regulations. Fermentation offers a green and sustainable option and has the potential application in industrial production. In the aspect of the milk globule, the HMFSs in infant formula still have some deficiencies compared to HMF, affecting the digestion efficiency of the lipid. With the full awareness of knowledge limitations, we hope to encourage additional research on HMFSs.

## Figures and Tables

**Table 1:** The total and sn-2 fatty acid composition of HMF [12,13,14,15,16].

Fatty Acid	Total Fatty Acid (%)	Sn-2 Fatty Acid (%)	Sn-2 Relative Percentage (%) *
C8:0	0.00–0.36	0.00–0.20	0.00–33.33
C10:0	0.15–3.10	0.21–1.60	8.25–33.33
C12:0	2.46–11.31	2.41–6.90	20.33–35.67
C14:0	2.46–11.63	6.20–15.40	37.58–57.97
C14:1	0.00–0.53	-	-
C15:0	0.09–1.11	0.46–0.53	72.09–78.47
C16:0	15.43–27.00	51.17–57.10	69.12–87.86
C16:1	0.00–3.60	1.60–3.50	14.81–38.99
C17:0	0.18–0.44	0.37–0.38	36.44–40.48
C17:1	0.10–0.34	-	-
C18:0	4.27–8.80	1.60–4.90	6.06–23.00
C18:1	28.30–45.88	8.10–17.43	7.89–14.18
C18:2	7.90–25.30	3.70–11.58	15.61–23.84
C18:3	0.00–1.50	0.28–0.90	15.15–26.30
C20:0	0.00–0.35	0.13–0.16	21.47–24.35
C20:1	0.23–1.68	0.40–0.51	17.41–22.06
C20:2	0.28–1.19	0.21–0.40	15.35–16.92
C20:3	0.25–1.57	0.25–0.34	16.66–20.28
C20:4	0.23–1.12	0.30–1.16	25.00–47.85
C20:5	0.00–0.24	-	-
C22:0	0.00–0.67	-	-
C22:1	0.00–0.66	-	-
C22:4	0.00–0.88	0.29–0.84	62.79–73.48
C22:5	0.00–0.22	0.27–0.33	66.92–72.29
C22:6	0.15–0.92	0.40–0.93	46.67–66.67

* Relative fatty acid in sn-2 position = (sn-2 fatty acid/3) × 100/total fatty acid in human milk. The sn-2 relative percentage data are calculated and concluded based on the data in the references.

**Table 2:** Fatty acid composition of PLs in HMF (%) [42].

Fatty Acid	PE	PI	PS	PC	SM
C10:0	-	-	-	-	0.1
C12:0	0.1–0.6	0.2–1.2	0.1–1.2	0.1–0.4	0.2–0.6
C14:0	0.2–2.4	0.4–3.3	0.1–2.5	0.9–4.5	1.1–2.1
C15:0	0.1–0.2	0.1–0.7	0.1–0.2	0.2–0.4	0.1–0.8
C16:0	7.2–11.8	5.8–17.3	7.3–13.4	25.1–38.0	5.3–21.3
C16:1	0.5–2.4	0.2–2.1	0.6–2.0	0.4–1.7	0.1–0.7
C17:0	0.2–1.5	0.2–0.7	0.6–1.0	0.3–0.7	0.5–1.4
C17:1	-	-	-	-	0.3
C18:0	23.1–29.1	30.6–34.5	33.5–42.8	16.9–24.7	11.8–13.8
C18:1	15.8–23.7	12.4–20.1	15.7–19.4	14.0–20.8	1.0–4.0
C18:2	13.0–23.8	5.3–19.5	8.5–23.0	13.9–24.1	0.3–4.5
C18:3	0.2–4.1	0.1–2.5	0.1–2.4	0.2–1.3	0.1–0.7
C19:0	-	-	-	-	0.4
C20:0	0.3–0.4	0.5	0.5	0.25–0.3	6.4–10.9
C20:1	1.3–1.4	0.2	0.5	0.4–0.7	0.1–0.5
C20:2	0.3–1.1	0.2–0.8	0.2–1.4	0.1–0.3	0.6
C20:3	1.1–3.5	2.0–5.2	1.3–3.9	0.6–2.4	0.2–03
C20:4	4.8–12.7	4.5–12.2	1.5–4.6	1.7–3.3	0.3–0.5
C20:5	0.3–4.2	11.7	0.5–9.0	0.1–2.9	0.2–5.3
C21:0	-	-	-	-	0.8–2.6
C22:0	0.2	-	-	0.2	12.9–20.7
C22:1	0.1–0.2	0.4	0.5	0.1–0.3	0.4–11.8
C22:2	1.5	-	-	0.1	4.8
C22:4	2.1–3.9	1.4–6.0	1.4–4.2	0.3–0.7	-
C22:5	0.8–2.4	0.4–2.2	1.6–3.0	0.4–0.9	-
C22:5	0.7–2.3	0.1–0.7	0.5–0.9	0.1–0.2	0.1
C22:6	1.0–5.1	0.4–1.7	1.5–2.9	0.1–0.6	0.5–1.1
C23:0	-	-	-	-	4.0–7.7
C24:0	0.3–2.8	0.9	1.2	0.1–0.5	8.1–19.5
C24:1	0.1–0.2	0.5	0.5	0.1–0.7	9.7–17.7

**Table 3:** Fatty acid composition of bovine milk fat and vegetable oils (%) [90,91,92,93].

Fatty Acid	Bovine Milk Fat	Coconut Oil	Palm Oil	Safflower Oil	Sunflower Oil	Soybean Oil	Canola Oil
C4:0	4.00–5.10	-	-	-	-	-	-
C6:0	2.10–2.90	0.00–0.04	-	-	-	-	-
C8:0	1.20–1.90	5.80–7.00	-	-	-	-	-
C10:0	2.40–3.50	4.80–8.00	-	-	-	-	-
C10:1	0.20–0.40	-	-	-	-	-	-
C12:0	3.00–4.10	48.00–51.02	-	-	-	-	-
C14:0	10.00–12.10	16.00–21.80	1.23–1.70	0.00–0.50	-	0.00–0.50	0.00–0.06
C14:1	0.40–1.30	-	-	-	-	-	-
C15:0	0.80–1.10	-	-	-	-	-	-
C16:0	28.70–34.10	8.40–9.20	41.78–43.30	4.00–7.50	3.70–6.90	9.00–14.50	3.75–10.50
C16:1	0.12–2.20	-	-	-	-	-	0.00–0.21
C17:0	0.40–0.50	-	-	-	-	-	0.00–0.04
C17:1	0.10–0.30	-	-	-	-	-	-
C18:0	10.30–13.30	1.94–2.80	3.39–4.80	2.50–2.70	1.98–2.90	4.00–5.20	1.87–6.90
C18:1	21.70–28.00	5.84–8.80	41.90–42.40	16.60–18.70	31.50–45.39	25.40–45.39	23.20–62.41
C18:2	1.50–2.30	0.50–1.28	7.80–11.03	71.10–76.00	46.02–59.50	46.02–51.90	15.20–20.12
C18:3	0.90–1.40	-	-	-	0.00–0.12	0.12–8.00	8.37–44.00
C20:0	0.20–0.20	0.00-0.25	-	0.00–0.20	0.00–2.30	-	0.00–0.64
C20:1	-	-	-	-	-	-	0.00–1.54
C20:2	-	-	-	-	-	-	0.00–0.11
C22:0	-	-	-	-	-	-	0.00–0.35
C24:0	-	-	-	-	-	-	0.00–0.27
C24:1	-	-	-	-	-	-	0.00–0.26

**Table 4:** Fatty acid composition of sources of LC-PUFAs used in infant formula (%) [94,95,96,97,98,99].

Fatty Acid	ARASCO	DHASCO	Tuna Oil	Cod Liver Oil
C8:0	-	-	-	-
C10:0	-	-	-	-
C12:0	-	3.60–4.40	-	2.21
C13:0	-	-	-	-
C14:0	0.34–0.58	18.50–19.40	3.27–3.42	3.83
C14:1	-	-	0.00–0.14	-
C14:2	-	-	-	-
C15:0	-	-	0.00–1.06	-
C15:1	-	-	0.00–0.09	-
C16:0	7.17–9.59	18.00–18.10	15.78–20.73	10.60
C16:1	-	1.80–2.00	4.14–6.14	6.97
C16:2	-	-	-	1.02
C17:0	-	-	1.39–1.58	-
C17:1	-	-	0.00–0.79	-
C18:0	7.70–10.50	0.40–1.00	4.52–5.89	2.73
C18:1	14.00–23.35	15.00–15.40	16.32–19.35	19.40
C18:2	4.56–7.62	0.60–1.00	1.35–1.84	1.43
C18:3	2.45–4.00	-	0.76–3.94	1.27
C18:4	-	-	0.00–1.23	2.29
C20:0	0.00–0.96	-	-	-
C20:1	-	-	0.00–1.76	9.40
C20:2	-	-	0.00–0.25	0.53
C20:3	0.00–4.30	-	0.00–0.31	0.47
C20:4	42.69–48.50	-	2.49–3.89	1.03
C20:5	-	-	6.35–7.62	8.89
C22:0	0.00–2.02	-	-	-
C22:1	-	-	0.00–0.93	7.57
C22:2	-	-	0.00–0.13	-
C22:4	-	-	0.00–1.20	0.50
C22:5	-	-	1.57–2.84	1.13
C22:6	-	38.40–39.00	22.85–26.86	10.70
C24:0	1.30–2.04	-	-	1.32
C24:1	-	-	0.00–0.77	-

**Table 5:** Studies of SL synthesis for infant formula.

Reference	Type of Reaction	Raw Materials	Solvent System	Lipase	Results
[129]	Acidolysis	Tripalmitin + caprylic acid	Hexane	Lipozyme^®^ TL IM (*T. lanuginosus* lipase)	Caprylic acid incorporation = 44.9 mol%
[140]	Acidolysis	Tripalmitin + oleic acid	Solvent-free	*Heterologous Rhizopus oryzae* lipase	Oleic acid incorporation = 22–30 mol%
[141]	Acidolysis	Tripalmitin + oleic acid	(1) Hexane (2) Solvent-free	(1) Lipozyme^®^ TL IM (2) Lipozyme^®^ RM IM	(1) Sn-2 palmitic acid = 92.92% OPO = 32.34% (2) Sn-2 palmitic acid = 86.62% OPO = 40.23%
[142]	Acidolysis	(1) Tripalmitin + oleic acid (2) Lard + oleic acid (3) Restructured palm oil + oleic acid	Hexane	*Candida lipolytica* lipase	(1) OPO = 46.5% (2) OPO = 45.0% (3) OPO = 32.4%
[143]	Acidolysis	Hazelnut oil + FFAs	Hexane	Novozym^®^ 435 (*C. antarctica fraction B* lipase)	n-3 PUFAs = 19.6%
[144]	Acidolysis	Microbial oil + oleic acid	Solvent-free	Lipozyme^®^ RM IM	Sn-2 ARA = 49.71% Sn-1,3 oleic acid = 47.05%
[145]	Acidolysis	OMEGA-GOLD oil + capric acid	Solvent-free	PS-30 lipase	Sn-2 DHA = 27.9% Sn-2 DPA = 12.6% Sn-1,3 capric acid = 13.3%
[146]	Batch acidolysis	(1) TGA40 oil + caprylic acid (2) TGA58F oil + caprylic acid (3) TGA55E oil + caprylic acid	Solvent-free	Immobilized *R. oryzae* lipase	(1) 1, 3-Capryloyl-2-arachidonoyl glycerol (CAC) = 36.0 mol% (2) CAC = 43.1 mol% (3) CAC = 50.7 mol%
[147]	(1) Acidolysis (2) Two-step acidolysis	Fungal oil + tripalmitin + oleic acid	Solvent-free	Lipozyme^®^ RM IM	(1) Oleic acid incorporation = 37.6% (2) Oleic acid incorporation = 55.4%
[122]	(1) Hydrolysis (2) Acidolysis	DHASCO + ARASCO + tripalmitin	Hexane	Lipozyme^®^ TL IM	ARA = 17.69%; DHA = 10.75%; Sn-2 palmitic acid = 48.53%
[148]	(1) Acidolysis (2) (2)–(7): Interesterification	(1) Tripalmitin + oleic acid (2) Palm oil + olive oil (3) Palm oil + camellia oil (4) Palm oil + earth almond oil (5) Lard + olive oil (6) Lard + camellia oil (7) Lard + earth almond oil	Isooctane	Lipozyme^®^ IM-20	(1) OPO = 55.2% (2) OPO = 21.8% (3) OPO = 25.2% (4) OPO = 19.9% (5) OPO = 12.9% (6) OPO = 15.4% (7) OPO = 9.0%
[149]	(1) Saponification (2) Fractionation (3) Esterification (4) Transesterification (5) Acidolysis	Oleic acid + refined palm oil	n-Hexane	Lipase IM 60	OPO = 74 mol%
[150]	Interesterification	Tripalmitin + ethyl oleate	Solvent-free	Lipozyme^®^ TL IM	Sn-2 palmitic acid = 80.6% Sn-1,3 oleic acid = 64.9%
[130]	Two-step interesterification	Amaranth oil + ethyl palmitate + DHASCO	Solvent-free	(1) Novozym^®^ 435 (2) Lipozyme^®^ RM IM	palmitic acid=33.9%; DHA = 1.9%
[134]	Esterification	DHA(by-product) + glycerol	Solvent-free	Immobilized *R. miehei* lipase	Sn-2 DHA = 17.3 mol% Sn-1,3 DHA = 51.7 mol%
[135]	Two-step esterification	Oleic acid + palmitic acid + glycerol	Solvent-free	Novozym^®^ 435	OPO = 34.98–39.55 wt.%
[139]	(1) Alcoholysis (2) Reesterification	DDD/EEE + ethanol + ethylcaprylate	(1) Ethanol (2) Solvent-free	(1) Novozym^®^ 435 (2) Lipozyme^®^ IM	1. 1,3-Dicapryloyl-2-docosahexaenoyl glycerol yield = 85.4% Regioisomeric purity = 100% 2. 1,3-Dicapryloyl-2-eicosapentaenoyl glycerol yield = 84.2% Regioisomeric purity = 99.8%
[151]	(1) Alcoholysis (2) Reesterification	Bonito oil +ethanol + caprylic acid	(1) Ethanol (2) Solvent-free	Novozym^®^ 435	(1) Sn-2 DHA MAG = 43.5% (2) CA-C22-CA = 51 wt.%
[137]	(1) Alcoholysis (2) Esterification	Tripalmitin + ethanol + oleic acid	(1) Organic solvent (2) Solvent-free	Immobilized lipase from *Rhizomucor miehei* and *Rhizopus delemar*	OPO yield = 78%
[138]	(1) Alcoholysis (2) Esterification	Tripalmitin + ethanol + oleic acid	(1) Acetone (2) Hexane	Novozym^®^ 435	OPO purity = 95% OPO yield = 90%
(1) [152] (2) [153]	Fermentation	Chemically interesterified fat/ mixture of ethyl oleate/ethyl palmitate	-	*Rhodococcus opacus*	(1) OPL = 40.09% (2) OPO = 47.1%

**Table 6:** Main composition of commercial OPO products.

Composition *	IOI Loders Croklaan		Advanced Lipids		Wilmar	
	Betapol^®^	Betapol^®^ Plus	InFat^®^	InFat^®^ Plus	Milkopas^®^ 9100	Milkopas^®^ 9320
Sn-2 PA (%)	55	65–75	52	52-	>52	>62
OPO (TG 52:2, %)	40–45	-	56–60	35–40	>40	>53
OPL (TG 53:2, %)	-	-	24	42	-	-

* PA: palmitic acid; OPO: 1,3-oleoyl-2-palmitoylglycerol; OPL: 1-oleoyl-2-palmitoyl-3-linoleoylglycerol.

**Table 7:** Legislated contents of fat in infant formula [185,186].

Component	Min	Max
Total fat (g/100 kcal)	3.3	6.5
Linoleic acid (g/100 kcal)	0.3	3.0
Alinoleic acid (mg/100 kcal)	50.0	-
Trans fatty acid (%)	-	3.0
Erucic acid (%)	1.0	2.0
ARA (%)	-	1.0
DHA (%)	-	2.0
Phospholipids (g/L)	-	2.0
Choline (mg/100 kcal)	7.0	50.0

## Data Availability

Not applicable.
